# Health Care Professionals’ Experiences of Facilitating Patient Activation and Empowerment in Chronic Illness using a Person-Centered and Strengths-Based Self-Management Program

**DOI:** 10.1177/17423953211065006

**Published:** 2021-12-14

**Authors:** Kristin Heggdal, Natalie Stepanian, Bjørg Frøysland Oftedal, Joshua B. Mendelsohn, Marie Hamilton Larsen

**Affiliations:** 1 Professor, 155319 Lovisenberg Diaconal University College, Lovisenberggata 15B, 0456 Oslo, Norway; 2 Assistant Professor, 5928 Pace University, College of Health Professions, Lienhard School of Nursing, 861 Bedford Road, Pleasantville, New York 10570; 3 Professor, 56627 University of Stavanger, 4036 Stavanger, P.O. box 8600, Norway; 4 Assistant Professor, 5928 Pace University, College of Health Professions, 163 William Street, New York 10038; 5 Associate Professor, 155319 Lovisenberg Diaconal University College, Lovisenberggata 15B, 0456 Oslo, Norway

**Keywords:** patient activation, empowerment, person-centered care, health intervention, self-management

## Abstract

**Objective:**

Patients with chronic illness who are empowered and activated are more likely to engage in self-management in order to stabilise their condition and enhance their quality of life. This study aimed to explore Health Care Professional's (HCP) assessment of a person-centered intervention called ‘The Bodyknowledging Program’ (BKP) for the facilitation of empowerment and patient activation in the context of chronic illness.

**Methods:**

This study employed a qualitative process evaluation after programme completion. Data was collected through focus-groups and individual interviews with HCPs and content analysis was used in the analysis.

**Results:**

Four themes were identified: 1) Shifts towards the patient-perspective, 2) The value of a patient-centered conceptual framework, 3) Patient activation through dialogue based support and 4) Challenging competencies. Discussion: This study introduces ‘The Bodyknowledging Program’ as a useful tool to uncover patients’ needs and to activate and empower them to take more responsibility for their health through self-care management. The usability of the new intervention depends on HCP's ability to develop their counselling skills and changing their approach towards utilising patients’ individual resources in the promotion of their health.

## Introduction

Improving and sustaining patient activation in patients with chronic illness is desirable because patients who are activated are more likely to engage in self-care behaviours that can improve their health and well-being.^1,2^ “Patient activation occurs when individuals believe they have an important role in their health and health care and have the knowledge, skills, confidence, and emotional commitment to perform this role” (p.1202).^1^ According to Wagner et al.^3^ “the patient with chronic illness must be the pilot of the plane”, that is; a knowledgeable and activated patient who functions as a collaborative partner in managing their health. While there is an increasing agreement that empowerment by way of patient activation is essential for improving quality of care and outcomes, programmes to support patients and HCP in these areas are few and not widely implemented.^4^ Interventions that combine person-centred care (PCC) and self-management support through interprofessional collaboration (IPC) report some degree of effectiveness in improving patient activation.^5^ PCC is based on the idea that patients are equal partners in planning, developing and accessing care.^6^ The purpose of PCC is to empower patients to improve and manage their health in accordance with their own beliefs, values and preferences.^7^

A study of health care professional's (HCP) experience of PCC at a multidisciplinary specialty clinic in Sweden reported that PCC led to a transformation of the roles of HCP's in patient meetings by encouraging more active involvement by patients and significant others and a more supportive role of HCP's.^8^ A prerequisite for successful PCC in clinical practice is to ensure that HCP have received adequate support and training.^9^ Nurses who had received training and support to practice PCC among people with type 2 diabetes in Scandinavia reported increased counselling competence and therefore, deeper insight into patients’ strength and vulnerabilities.^10^ HCP's treating people with progressive neurological conditions in England conveyed that training in health coaching methods was useful; however, it was difficult to fully embrace the coaching role in settings where their clinical experience was more highly valued.^11^ This aligns with other studies reporting difficulties for HCP in moving out of their professional disease-focused treatment perspective towards emphasising the person's lived experiences of illness and utilising personal resources for health.^12^

Alvarez et al.^13^ found that levels of patient activation were positively correlated with clinicians’ assessment regarding the patients’ role in self-management. However, the traditional roles of HCP are deeply embedded in the culture of medicine and often inconsistent with empowered and activated patient roles within an equitable and collaborative therapeutic partnership with HCP.^1^ In order to promote the health and longevity of patients with chronic illness, interventions are needed that combine person-centeredness, empowerment and activation of patients’ capabilities for health. When interventions have clear concepts and are theory-driven, these factors might increase the likelihood of successful translation into practice.^14^

Led by interdisciplinary HCP, the Bodyknowledging program (BKP) is an example of a theory-based intervention that has been broadly applied across diagnosis, age, gender and clinical settings in Norway.^15^ Patients with various chronic illnesses and multidisciplinary HCP were involved in the development and testing. Significant changes in recovery and health were demonstrated in patient-reported outcomes.^16,17^ Although the theoretical model underpinning the BKP posted patient activation as a key construct for explaining patient outcomes, HCP facilitators' views on the execution of this construct in practice were uncertain. This study aims to explore HCP's assessment of the BKP as an intervention that facilitates patient activation and empowerment in chronic illness.

## Methods

### Study design

The present analysis was part of a larger study in which the BKP intervention was piloted in different clinical sites. In line with the recommendations for the development of complex interventions, a qualitative process evaluation was undertaken to identify key components and the connections between intervention activities and outcomes. The method also served to examine the applicability of the theory underpinning intervention design.

### The Bodyknowledging program (BKP)

Bodyknowledging theory served as the theoretical framework for the intervention.^18^ The theory posits that people diagnosed with chronic illness have inherent resources for coping and health. Specifically, their knowledge of the type and magnitude of activity and factors within their physical and psychosocial environment is an essential, underutilised, resource. The programme also draws on Merleau-Ponty's phenomenological theory^19^ of the body as a foundation for knowledge and existence, and Antonovsky's^20^ theory of health as a dynamic continuum. The pedagogical approach of BKP was inspired by Paulo Freire's theory of empowerment^21^ and the philosophy of active learning,^22^ which has demonstrated that people are able to cope with life and learn the most when they are involved and activated. The BKP presents phases of patient-centered expertise for interpretation and application by intervention participants. This approach is in keeping with Paulo Freire's^21^ “pedagogy of the oppressed” in which the person in question defines their situation and dialogue serves as the main method for helping people to understand their situations and to act in new ways. The BKP seeks to engage patients by revealing their own bodily knowledge as a resource for health. It presents “health” as a process to be strengthened by their own efforts through encounters with their environments. Varied methods such as dialogue, group work, physical activities and diary writings are used to facilitate their health promoting process.

### Organisation of the intervention

The intervention is organised into seven sessions (Table 1). The first three sessions are held weekly; the next three every second week. The final, seventh session is scheduled 3.5 to 4 months after the programme start date. Individual sessions last 1.5 h. Group sessions of 8–10 people with various health conditions are 3 h with a 30-min break to eat and socialise. Nurses, occupational therapists, and physical therapists complete 80 h of training (Table 2) in leading the intervention.

**Table 1.** Structure and content of The bodyknowledging program.

Structure	Content, pedagogical methods and tools	Purpose
**Week 1:** Programme Introduction Session 1.	Information about programme structure, content, and pedagogical tools. Participants’ expectations of the programme. Short introduction to the Bodyknowledging Model.	Provide information to enhance security and predictability for participants. Creating a safe environment for each person to engage in the programme. Establish trust and dialogue with patients as equal partners in health promotion.
**Week 2:** Uncertainty (introduction) Session 2	Participants' own themes about living with chronic illness. Studies of patterns of former patients' experiences of each phase of the Bodyknowledging process, especially the phase of uncertainty - escaping and denying the sick body. Introduction to physical exercises inspired by Body Awareness Therapy. Introduction to the use of the booklet/diary between sessions.	Encourage patient participation and motivation to engage in the programme. Through the exercises, help the participants to attend to their body as a valuable resource for coping and health.
**Week 3:** Loss of Life Space (introduction) Session 3	Participants' own themes concerning challenges connected to living with a long-term condition and their health promotion strategies. Introduction to the phase of Losing life space - grieving and anger. The use of booklet/diary between and in sessions. Physical exercises.	Facilitate dialogue and reflection work on each participant`s process of health promotion. Allowing patients to express themselves and their experiences of their reactions and challenges involved in living with health problems long-term.
**Week 5**: Loss of Life Space (in-depth Work) Session 4	Participants' own themes and strategies concerning health promotion in chronic illness. Introduction to the phase of Listening and understanding the body's signs - strengthening hope. Acknowledging their body as a source of knowledge about health. Communicating their limits of tolerance to others. The use of booklet/diary. Physical exercises.	Support the participants to utilise their bodily knowledge as a resource to promote their health, i.e. helping them to discover their limits of tolerance concerning type and magnitude of activity.
**Week 7:** Listening and Understanding the Body's signs (introduction) Session 5	Participants' own themes and strategies for creating health within illness. How to get to know one's limits of tolerance for activity, factors of the physical and social environment. Encounters with others. The use of booklet/diary. Physical exercises.	Strengthening the participant's ability to set limits for themselves and others in accordance with their body's limits of tolerance in order to stay healthy. Training in handling the reactions of others.
**Week 9:** Listening and Understanding the Body's signs (in-depth Work) Session 6	Participants' own themes of health promotion in chronic illness. Introduction of the phase of Integrating embodied knowledge - exploring new possibilities for health. Encounters with society. The use of booklet/diary. Physical exercises.	Motivation to explore their body's dynamic limits of tolerance and their possibilities for activity and lives unfolding within these limits.
**Week 12:** Integrating Knowledge – New Possibilities for Well-being and Health Session 7	Summary of the Bodyknowledging Model in relation to each participant. Encounters with society. Physical exercises. Participant's` thoughts and actions on how to sustain and strengthen their health after programme completion.	Encourage and help participants to make decisions on how to sustain their health and recovery, and how to sustain or enhance their participation in society.

**Table 2.** Training program for health care professionals (HCP) leading the bodyknowledging program (BKP).

Course 1. Health promotion processes	Content
TWO 3-day meetings (1in-person and 1online) **Method of work:** Resource lectures are arranged based on the central topics. Written report 1 for study group with oral presentation during video conference. Written report 2, individually, with guidance from supervisor.	**Fundamental perspectives in health promotion work** The resource perspective in meeting with people who live with health problems or who are at risk of being afflicted. The Salutogenetic theory, Theory of coping, and the application of the theory in practice. **Health-promotion processes** Recovery research on long-term illness within somatics and psychiatry. The health-promotion process: Bodyknowledging and patients/users' experiences with health-promotion processes. **Empowerment and user participation** The concepts of empowerment and user participation. Patients as experts on their health and how to handle health problems. Tools and making accommodations for user participation in practice. **The significance of social relations for coping and health** Social networks and social context; inclusion and social interaction that promotes healing. **Health education** Dialogue as a fundamental approach in health promotion work. Narrative method, storytelling and writing as a method in health education. Group methods and group processes.
**Course 2: Bodyknowledging as a process-oriented approach to coping and health**	**Content**
THREE 3-day gatherings (1 in-person; 2 online) **Method of work:** Written report 3: Plan for carrying out the BKP in practice. Practical training on implementation of the BKP. Written report 4: After meeting with patient/group Individual process reports from practical training. Instructor evaluation of competencies.	**Bodyknowledging as an educational health system and tool for health promotion.** Structure, contents, methods and tools of the BKP. Resource use and requirements for organization and quality. Interdisciplinary collaboration. Documentation of change throughout the process. Reflection upon own practice. BKP as an interactive tool and follow-up program in the specialist and municipal health services. BKP organized as a coping course, tool for individual tailoring of care and prevention, rehabilitation measures and preventive measures. BKP compared with other educational health systems. **Health education** Solution-focused approach to health promotion work. The physiotherapeutic method of Basic Body Awareness (BAT). Dialogue, story-telling, writing, solution-focused therapy and exercises inspired by the physiotherapeutic method of BAT.

Two HCPs lead each group; one HCP leads each individual sessions. A poster, a flip-over chart, and a booklet/diary that contains an overview of the Bodyknowledging process, served as pedagogical tools. Sessions begin with 15 min of physical exercise focused on breathing, balance and movement. The group leaders used the poster to introduce the Bodyknowledging process. Beginning with a short overview of the process in the first session, phases are introduced one by one in the following sessions. Using the BKP framework^18^ as a background, participants are invited to reflect on how they experienced their health and how, in their life situation, health and well-being could be enhanced. Group leaders emphasise dialogue by posing open-ended questions and by offering time for each person to express their views. The questions are printed on the flip-over as well as in the booklet/diary, which also contain statements from patients. A booklet on Bodyknowledging (with questions) serves as a guide to personal work on recovery. Participants were encouraged to read the booklet at home, to reflect on the questions posed, and to write about their experiences. At the next session, participants are encouraged to share their reflections on health-related challenges, explore their recovery strategies and engage in the group process. In this way, participants’ own life situations, coping strategies, and health-promoting abilities form the core of the programme's content. In addition, patients choose a physical activity to do at home twice a week. Details on the intervention development and components have been published elsewhere.^15^

### Sample, study setting and recruitment

Eight HCP were selected purposively in order to include interdisciplinary perspective (i.e. nurses (n = 4), occupational therapists (n = 3) and 1 physical therapist). Their professional experience varied from 5–35 years. They represented three different clinical sites: a rehabilitation unit, an outpatient clinic, and a hospital-based centre for patient education, known as a “Learning and Mastery Center” (LMC) (Table 3). The HCP led the intervention in pairs with 8–9 patients diagnosed with a variety of chronic illnesses (Table 4). Two groups were situated at the rehabilitation unit in one hospital and two groups at the LMC (n = 38 patients were involved in group sessions). The individual format was led by a nurse at the rehabilitation unit in one hospital and another nurse at the other hospital's outpatient clinic (n = 20 patients in individual format). One of the nurses could not complete the training and data from her pre-training was excluded from the analysis. Results from patient interviews have been published elsewhere.^16,17^

**Table 3.** Health care professionals by gender, age, and clinical site.

Participants	All HCP (N = 8)	Nurses (N = 4)	Occupational Therapists (N = 3)	Physical Therapist (N = 1)
Men	0	0	0	0
Women	8	4	3	1
Age range	36–60	36–55	44–47	60
Years of experience	5–35	10–30	5–20	35
Rehabilitation Unit (Inpatient), Hospital 1	5	2	2	1
Outpatient clinic, Hospital 2	1	1	0	0
Learning and Mastery Center (Outpatient clinic), Hospital 2	2	1	1	0

**Table 4.** Patients by gender, age, diagnostic categories, and site (N = 58).

	Group 1 LMC (N = 11)	Group 2 LMC (N = 9)	Group 1 Rehab (N = 8)	Group 2 Rehab (N = 10)	Individual format Rehab (N = 13)	Individual format Outpatient Clinic (N = 7)
Men	6	3	8	6	9	2
Women	5	6	0	4	4	5
Age range	56–68	29–69	48–89	46–66	23–81	31–64
COPD (N = 4)	2	2				
IBD (N = 8)		1				7
Stroke (N = 18)			6	3	9	
Multiple Sclerosis (N = 2)				2		
Neurological disease (N = 1)				1		
Head injury (N = 1)			1			
Brain Tumor* (N = 2)				1	1	
Cerebral atrophy (N = 1)					1	
Leg amputated (N = 3)			1	2		
Spina bifida (N = 2)				1	1	
Musculoskeletal pain (N = 5)	1	3			1	
Heart disease (N = 4)	4					
Diabetes (N = 3)	2	1				
Depression (N = 1)		1				
No diagnosis (N = 3)	2	1				

LMC: Learning and Mastery Center, COPD: Chronic Obstructive Pulmonary Disease, IBD: Irritable Bowel Disease *Rehab after surgery.

### Ethical considerations

The ethics committee of the Southeastern Regional Health Authorities in Norway approved the study (REK number:1.2005.2746) and all the participant received information on the purpose and method of the study and their right to withdraw from the study at any time. All HCP facilitators (n = 8) who led the intervention, volunteered to participate in evaluation interviews and signed an informed consent form.

### Data collection

Qualitative data was collected by means of focus-group and individual in-depth interviews with HCP using a semi-structured interview guide. Key discussion questions asked about HCP`s assessment of the BKP was: How was your experience of leading BKP groups? How did the content, the pedagogical tools and approach work? What were the results for you as a professional in leading the BKP programme?

In order to ensure saturation, the first author conducted four focus group and two individual interviews 1–2 weeks post-intervention. With participant consent, all the interviews were audiotaped and transcribed verbatim. One focus group (n = 5) was conducted at the rehabilitation unit and one was conducted at the LMC (n = 2). Two focus-groups (n = 8) were conducted collectively with all HCP participants from all three sites. Finally, two individual interviews were conducted with one nurse who worked individually with patients in the outpatient clinic. Transcripts were checked for accuracy prior to analysis.

### Analysis

The first author and a scientific assistant analyzed the data from each site independently and discussed the preliminary findings. Then, the data were analyzed across sites. To ensure confirmability and dependability, findings were discussed with the research team. The evaluation focused on the HCP's assessment of patient outcomes in relation to intervention activities and outcomes for them as professional's. Qualitative content analysis was used to analyze the data. The parts of the text that described the HCP's assessment of outcomes were extracted and the text was divided into meaning units that described similar content, abstracted into themes and subthemes, then labeled with a code. Themes, subthemes, and codes were sorted, discussed, and studied again to develop overarching themes. By analyzing the findings across interviews, we identified the features of how and why the programme functioned across patient groups and research sites.

## Results

HCP described that the BKP represented something new that allowed them to work together while being focused on the patients’ capacity to promote their health within a specified timeframe.

The analysis established four themes that describe HCP's assessment of how the BKP promoted patient activation by: 1) Shifts towards the patient-perspective, 2) The value of a patient-centered conceptual framework, 3) Patient activation through dialogue based support 4) Challenging competencies.

### Theme 1: shifts towards the patient-perspective

The HCP describe that the BKP had introduced a major change in their professional role. This involved a shift from emphasising disease management towards emphasising the patient perspective on how best to utilise their capacity for health and recovery through enhanced activation. This shift was facilitated by the grounding of the intervention theory in patients’ understanding and articulation of health within illness while living with a chronic illness:

The patients’ expressions on how to recover in different phases in their illness trajectory is pervasive in every part of the theory and in the process tools of the program. It is the user's words and language that dominates. When we have this model and process tools, we have a quite different and new possibility to handle the challenges that patients bring up. We have a unique possibility to strengthen their resources and effort to stay well as they live with chronic illness (OT).

This changed attitude and approach allowed HCP to consciously acknowledge the patient perspective by facilitating the expression of their experiences and concerns, taking the patient's perspective as a point of departure, and strengthening the patient's capabilities to handle their health problems and to utilise their inherent health resources. The HCP argued that this level of openness to patient experience, while keeping their professional instincts in check, required a challenging level of humility, self-control and acknowledging a new type of collaboration with patients. This humility required practitioners to develop the competence to balance active listening and “holding back” on the routine of giving advice. At the same time, practitioners found that they developed new skills to absorb signals from patients concerning their standpoint and health-related needs. The HCP's shift of perspectives towards emphasising the patient perspective and the attitude of equality was described as a “door-opener” for patient activation.

### Theme 2: The value of a patient-centered conceptual framework

Healthcare professionals describe that the BKP's theoretical framework implies a new way of thinking and approaching people with chronic illness as knowledgeable, capable, and resourceful. The framework were integrated in the intervention structure and the pedagogical tools. According to HCP, this functioned well in clinical practice when it came to supporting, challenging, and confirming patient engagement in taking charge of their health. HCP reported that patients participating in BKP added to the intervention's content by sharing their story in support groups. HCP described how patients were assisted in their movement towards better health by reflecting on the conceptual framework:

The model stimulates the patients’ associations, and the aspects or parts of the model that create recognition, activates them and then, we can help them to move further on by facilitating their individual process. This motivates and creates movement. When a patient says something, I sometimes ask the group if they have similar examples, and then, their experiences are being confirmed. I think this creates some sort of community in the group. Then, we often link these experiences back to the model to place their experiences in a bigger frame and to work more in-depth with their process (RN).

The findings indicate that the theory itself creates a focal point for expressions of support for BKP participants as it reflected former patient's experience of handling the challenges of chronic illness. To this end, patient activation was strengthened, according to HCP, by utilising the concepts in the programmes’ content and pedagogical approaches.

The patients feel that they are co-operators and have an impact on their treatment, and they are very happy when we believe in them, that we rely on their experience and trust their knowledge of their body and illness (PT).

These findings indicate that HCP perceive that the BKP facilitates a new patient role in the sense that patients are challenged to take on a much more activated role in relation to their own health and are supported as they engage with this opportunity.

### Theme 3: patient activation through dialogue based support

HCP reported that they had learned new ways of communicating with patients through the BKP training that had consequences for their approach and dialogue with both BKP participants and patients in general. The dialogue aimed to support patients in doing the “research” on what makes their condition better or worse, that is, factors that they can self-regulate like type and amount of activity and type and number of modifiable factors situated within their environment.

HCP reported that it was novel for them to invite patients to reflect on their strategies and require them to be active in the group work, to ask them to do some ‘homework’ like reading the booklet, answering questions posed in the booklet and doing physical exercises between sessions. The approach represented a change in their professional role and involved a new kind of trust in the patient related to accepting patient input as a valuable asset to inform HCP treatment plans and decisions. HCP reported that this change facilitated a level of comfort in the patient-provider relationship that allowed them to productively activate the patient to engage in health promotion by posing questions like: What can you do to prevent a new relapse and to strengthen your health capacity? What support do you require from your significant others and from us to do so? This way of posing questions was a new skill that they deployed in their routine patient contacts.

I did not ask such questions that we use in BKP a year ago, no. Then, I focused strictly on giving information, but also on caring and supporting for the patient of course. I still inform about the facts that it is not only easy to live with chronic illness and to express that I understand, but I think that the new approach makes it easier for patients to express their needs when it comes to other aspects than medication (RN).

This approach involves moving beyond a uniquely medical focus towards a more holistic approach to health by activating the whole person and harnessing their capabilities for health. According to HCP, this became a major focus of the patient-provider dialogue.

### Theme 4: challenging competencies

HCP described their experience of the BKP intervention as a learning opportunity that promoted skills development in how to approach patients diagnosed with chronic illness, while also taking up a new professional role by developing and integrating new competencies within their routine practice. The analysis revealed that this was a challenging experience as it implied moving out of their comfort zone while developing new competencies.

It has been fantastic to be a part of this and a privilege to be asked to participate. I am very humble with regards to all the work that is laid down beforehand. At the same time, my motivation has varied because I think it has been a bit demanding and I have been afraid of not doing a good enough job because there is always something you could have done better. I had some sort of performance anxiety, and I was stressed because we did not manage to follow everything according to the manual. We had to make some parts shorter and allow the patients to get more time to express themselves and to take what came up spontaneously (RN).

HCP described that the training in BKP, theory and new pedagogical tools led to a substantial change in practice that required new competencies and skills when it comes to their encounters with patients. This included, for example, the ability to “hold back” on their interpretation of the patient's situation and their eagerness to give advice, and the ability to wait, listen and receive the patient's description of their situation and then to ask follow-up questions. HCP expressed that it was challenging for course leaders to attain good communication skills in order to practice a person-centered approach that strengthened empowerment and patient activation.

As a course leader, you cannot be ‘trapped in your own world’. You have to be able to capture what's out there and not necessarily follow the recipe but be capable of letting the conversation in the group unfold at the same time as one tries to link back to the main theme of the session. This is important, and to do it without pushing something on them (OT).

HCP emphasised the importance of the course leader's openness and professional competence as a key to productive group work with patients. However, sometimes it was difficult to be a course leader, i.e. when the openness in the group led to sensitive revelations like interpersonal violence or abuse, or other issues that fell outside the course leaders’ competencies. Setting the boundaries while attending to the participants’ individual needs that arouse from these disclosures was challenging.

HCP emphasised the opportunity for improving their competence in leading a group of patients in self-care and the advantage of working with colleagues who had different kinds of professional training and experience, and a longer track record in group work. According to HCP, the way they had worked together built their individual competencies as course leaders for patients with chronic illness, while also strengthening the IPC.

The Bodyknowledging theory and program is an important interdisciplinary tool that allows us to move together in “the patients’ world”. In addition, the BKP offers us shared concepts, and a shared platform and theory across professional groups in order for us to encounter the users in a coherent and good way. In this way, we can complement each other's contribution by means of the professional strength that each of us have (OT).

According to HCP, one strength of BKP is the comprehensiveness, that many aspects of living with chronic illness are included; while content that is grounded in a theory that support skills normally difficult for a health care service to provide, i.e. the focus on the patients inherent resources and capabilities for health (i.e. bodily knowledge of limits of tolerances for activity, social relations and so on). This was new to HCP, and a tool to uncover patients’ needs in support of activation, empowerment and follow-up. The BKP's structure and pedagogical tools served to confirm, support and motivate the patient to trust their own experience-based knowledge and to take more responsibility for their health.

## Discussion

This study aimed to explore HCP's assessment of the BKP as a innovative intervention that facilitates patient activation, empowerment and PCC in chronic illness. The findings revealed the value of the underlying theoretical approach which allowed HCP to develop a shared de-medicalised frame of support that worked well in facilitating empowerment and patient activation for self-management in patients with chronic illness.

According to Lin et al.,^23^ HCP should implement patient activation interventions that strengthen the patients' role in managing their healthcare to improve health outcomes as there is strong evidence that interventions focused on patient activation can improve patient health as measured by physiological, psychosocial, and behavioural outcomes. In respons, we examined the experiences of HCPs in delivering a person-centered intervention to evaluate the process of HCP engagement with patients and intervention activities, and perceived effect on patient activation.

Upon reflecting on their implementation of the BKP intervention, HCPs had developed their competencies. They reported that the BKP required them to develop new competencies in relation to attitudes and approaches to their patients. Findings from a large international study^24^ that aimed to describe HCPs perspectives on how to improve the provision of person-centered diabetes care found that HCPs debated whether listening to their patients was an important strategy in diabetes self-management; however, they struggled to make the transition from those who “tell” to those who “listen”. In contrast, our work suggests that upon engaging directly in a PCC-focused intervention, HCPs reported that their understanding of how best to deliver care shifted to a more patient-centered framework. According to HCP in this study, a structured intervention using a patient-centered and empowerment- based theoretical framework, made this transition easier and promoted positive collaboration with patients and colleagues. These benefits were valued by HCP. This positive effect on IPC noted in a study by Alvarez and colleagues^13^ showing that HCP who scored high on a measure of clinician support were more likely to engage in patient-centric approaches to self-management and behaviour change. Importantly, levels of patient activation were positively correlated with clinicians’ assessment of the patients’ role in self-management.

Patient activation requires the HCP to incorporate a person-centered approach, therefore HCP require adequate support and training in the use of person-centered principles in practice.^9^ HCPs in our study reported developing a shared frame of reference with their patients that enabled them to work more productively as partners on promoting their patients’ health. This shows how user involvement in BKP transformed the relationship between patients and HCPs for the better. According to Greene and Hibbard,^4^ programmes to support patient activation and engagement are not yet well developed or widely implemented, which is a potential pitfall as HCP seek to improve quality and decrease health costs. Patient activation was not measured in our study, however, HCP reported that BKP strengthened patients comprehensive understanding of their health and their use of inherent resources.

If patient activation is a self-management imperative, it is important that HCP themselves are willing to change. However, this is a challenge given their training and expert role and requires a shift from their professional disease-focused, treatment perspective towards an emphasis on an equitable collaborative partnership with patients that aims to facilitate personal capabilities.^12^ Working along the lines of empowerment and patient activation in chronic illness implies supporting the patient ‘to become the pilot of the plane’.^3^ Understanding the value of the patients’ bodily knowledge of health and illness, and emphasising their capabilities for health and recovery is an essential part for this change.^18^ Even if the paradigm shift from paternalism to patient involvement was acknowledged some decades ago, it continues to be challenging to implement in practice. There is a need for interventions like the BKP to assist HCP to relinquish power for the purpose of initiating and sustaining patient empowerment and activation.

The interprofessional HCPs in this study, perceived the BKP as a tool to utilise patient's expertise within patient-centered care and to acknowledge the patient's capability of making informed decisions regarding self-management in chronic illness.

Upon completing their facilitation of the BKP programme, HCP stated that they had improved their communication skills and were much more reluctant to give quick answers and advice. Their new competencies include an aptitude for asking questions and challenging patients to take more responsibility for their health and to actively engage in their treatment and care.

Qualitative research regarding how patients perceive and communicate about what is important to them in their communication with HCP has shown that some patients may avoid speaking about sensitive topics or on issues they suspect that their views might conflict with those of their HCPs.^25^ An additional common communication barrier was the time limitations of clinic visits, which prevented them from sharing details about what was important to them. Our findings show that trust and dialogue built up by meeting patients in an informal intervention context where active listening and empathy was fostered, facilitated better communication with patients from a HCP perspective.

While participating in the BKP as a facilitator led to the development of new skills in treating patients diagnosed with chronic illness, this also felt challenging to HCPs, as it involved moving out of their professional comfort zone and developing new competencies. Similarly, in a study of diabetes specialist nurses, Boström et al.^9^ found that efforts to motivate patients to self-manage their condition were perceived as time-consuming for the staff. In contrast, we found that a structured, theory-based and PCC intervention (BKP) focused on enhancing patient activation and empowerment, resulted in reports by HCP of a reorientation in their focus towards their patients through enhanced trust and dialogue. This response was facilitated by tools for IPC. HCP held the view that their experience with BKP had built a new foundation for the different professional groups to support the patients' as a team. This aligns with a study of HCP experience of PCC at a multidisciplinary specialty clinic in Sweden where PCC led to a transformation of the roles of HCP's in patient meetings by encouraging more active involvement by patients and significant others and a more supportive role of HCP's.^8^ Drawing on Freire,^21^ a basic tenet of the BKP approach is that solutions are to be found in each person's realisation of their life situation, in the sense that individuals need to define their situation and take “ownership” if empowerment is to take hold. Our findings revealed that the BKP established a change in HCP's way of thinking and in their approach towards patients that facilitated patient activation and empowerment to self-manage chronic illness.

## Strengths and limitations

One strength of this study was the diversity of HCP and clinical context. In addition, HCPs experience involved the use of BKP in encounters with a variation of chronic illnesses. Focus group interviews and individual interviews together served to establish a rich dataset. An inherent limitation of focus groups, is that data collection is based on the social interaction and “knowledge construction process” in the group and hence, may not represent the individual views of participants. This limitation was mitigated by means of validation in individual interviews and by conducting interviews across sites. HCP participating in this study took the initiative to develop the intervention, participated in the formative research and engaged in evaluation interviews. While a high level of clinical relevance was apparent, HCP participated in the evaluation of their own work with patients, which may have biased their reports of patient activation.

## Conclusions

The findings of this study indicate that a structured theory-based programme was an advantage for HCP who wanted to orient their clinical practice towards improvements in patient activation, empowerment, and self-management of chronic illness.

The establishment of an atmosphere of safety and trust in patients' capabilities to take charge of their health was important for HCP in delivering the intervention and anchoring IPC.

## Practice implications

Continuing education in the basic tenets of empowerment, PCC and patient activation is needed in order to ensure improvement in chronic care practice. Online training courses in the BKP should be developed in order to make it accessible for different contexts and sites. Future work should build on the findings of this study while adapting and testing the intervention in novel contexts.
